# Post‐dialysis recovery time in ESRD patients receiving more frequent hemodialysis in skilled nursing facilities

**DOI:** 10.1111/hdi.13012

**Published:** 2022-04-06

**Authors:** Eran Y. Bellin, Alice M. Hellebrand, Steven M. Kaplan, Jordan G. Ledvina, William T. Markis, Nathan W. Levin, Allen M. Kaufman

**Affiliations:** ^1^ Departments of Epidemiology & Population Health and Medicine Albert Einstein College of Medicine Bronx New York USA; ^2^ Dialyze Direct Neptune City New Jersey USA; ^3^ WTM Consulting Passaic New Jersey USA; ^4^ Internal Medicine Mount Sinai Icahn School of Medicine New York New York USA

**Keywords:** ESRD, home hemodialysis, more frequent dialysis, mortality, nursing home, post‐dialysis recovery time

## Abstract

**Introduction:**

Post‐dialysis recovery time (DRT) has an important relationship to quality of life and survival, as identified in studies of ESRD patients on conventional dialysis. ESRD patients are often discharged from hospitals to skilled nursing facilities (SNFs) where on‐site treatment using home hemodialysis technology is increasingly offered, but nothing is known about DRT in this patient population.

**Methods:**

From November 4, 2019 to June 11, 2021, within a dialysis organization providing service across 12 states and 154 SNFs, patients receiving in‐SNF, more frequent dialysis (MFD) (modeled to deliver 14 treatment hours minimum per week and stdKt/V ≥2.0) were asked to describe their post‐dialysis recovery time following their previous treatment, within predefined categoric choices: 0–½, ½–1, 1–2, 2–4, 4–8, 8–12 h, by next morning, or not even by next morning. Patients reporting DRT following at least one full‐week treatment opportunity were included in a mixed model logistic regression of rapid recovery (DRT ≤2 h).

**Findings:**

Two thousand three hundred and nine patients met the statistical modeling inclusion criteria, providing DRT on 108,876 dialysis sessions, while receiving mean (SD) 4.3 (0.96) weekly dialysis treatments. 2118 (92%) reported DRT ≤2 h. Results appeared biologically plausible, as lower odds of rapid DRT were observed for patients who were older, missed their previous treatment, or experienced intradialytic hypotension. Greater odds of rapid DRT were observed in patients receiving five dialyses in the previous week or having 160–179 mmHg pre‐hemodialysis systolic blood pressure. Rapid recovery was associated with reduced mortality or hospitalization.

**Discussion:**

SNF dialysis patients receiving 5x per week MFD report rapid recovery time ≤2 h in 92% of dialyses despite advanced age, frailty, and comorbidities. Future studies will assess the practical ramifications of rapid DRT perception/experience on nursing home rehabilitation programs, which could impact patient health beyond the nursing home stay.

## INTRODUCTION

Patients with end‐stage renal disease (ESRD) receiving chronic hemodialysis (HD) often experience a prolonged, debilitating period of post‐dialysis downtime and recovery following treatment. The duration of this period, known as post‐dialysis recovery time (DRT), has been characterized through a validated patient questionnaire.^1^ In the general ESRD population receiving conventional dialysis, DRT is inversely correlated with multiple quality of life and physical symptom assessments,^2^, ^3^, ^4^, ^5^ and several studies document more than half of patients with DRT greater than 2 h.^6^, ^7^, ^8^, ^9^ Prolonged DRT is associated with increased hospitalization^6^, ^10^ and mortality.^6^ A more frequent dialysis (MFD) treatment schedule has been associated with improvements in patient quality of life and laboratory measurements,^11^ and in a randomized controlled trial, six times per week MFD compared to conventional dialysis resulted in improved fluid management^12^ and reduced hospitalization rates^13^ and mortality.^14^ Two prospective studies^1^, ^15^ and a randomized controlled trial^4^ showed that switching to daily MFD results in a significant DRT reduction.

In 2016–2018, 6.5% of incident ESRD patients resided at least transiently in a skilled nursing facility (SNF)^16^—a likely underestimate,^17^—and this figure should continue to increase in an aging population. Dialysis patients admitted to SNFs are characterized by advanced age, frailty, and multiple comorbidities, and many of them require assistance with daily living.^18^ Many of these patients are transient residents admitted to the SNF for subacute rehabilitation after an acute hospitalization,^18^ and a prolonged DRT is a potential barrier to successful rehabilitation programs. Increasingly, MFD is provided to the SNF HD population,^18^ based on its aforementioned benefits found in the general dialysis population.

Nothing is published about post‐dialysis recovery in SNF‐resident ESRD patients. We sought to characterize DRT in such patients. We hypothesized that provision of MFD would be associated with relatively short recovery times.

## MATERIALS AND METHODS

### Setting

ESRD Patients residing in SNFs received on‐site hemodialysis with MFD treatment schedule targeting a minimum of 14 h of treatment per week and std Kt/V ≥2.0, provided via NxStage System One technology and pursuant to a physician's order. Care was provided by Dialyze Direct™ as described previously^18^; a specialized and regionally coordinated dialysis nursing staff operated on‐site in the SNF to deliver treatment. In the present study, patients were treated in 154 nursing homes across 12 states (Florida, Illinois, Indiana, Kentucky, Maryland, Missouri, New Jersey, New York, Ohio, Pennsylvania, Tennessee, and Texas).

### Subject constructs

#### Dialytic episode

The dialytic episode is defined as the interval starting from the patient's first in‐SNF dialysis session, inclusive of all subsequent dialysis sessions, until treatment cessation at that facility due to hospitalization, death, transfer to another facility, transfer to home, or withdrawal from therapy. Upon a readmission to the SNF, the first dialysis session following readmission initiates a new dialytic episode.

#### Post‐Dialysis recovery time (DRT)

Beginning on November 04, 2019, Dialyze Direct initiated a company‐wide effort to collect DRT information. At each dialysis session, the patient was asked the duration of recovery time to baseline function following their antecedent dialysis session with specific categoric answer choices: 0–½, ½–1, 1–2, 2–4, 4–8, 8–12 h, by next morning, or not even by next morning. Nurses recorded whether the patient was unable to respond due to cognitive impairment or physical inability to speak including intubation.

### Data sources and group designation

Relevant demographics, clinical care, and other dialysis information was obtained through a retrospective study of all dialysis care data recorded by Dialyze Direct from November 4, 2019 through June 11, 2021 in either of two electronic medical records (EMR), GAIA (Gaia, Littleton, CO), and Clarity (Visonex). Clarity was readily amenable to the addition of the recovery time survey, and the DRT question was posed only to those patients in facilities utilizing Clarity. All DRT data recorded by Dialyze Direct were obtained from Clarity.

The DRT cohort consists of all patients who could provide a report of their recovery time following at least one complete Monday through Sunday interval of a dialytic episode. We required a week of treatment opportunity to evaluate the effect of the SNF dialysis routine on the patient's recovery time and to minimize the influence of acute hospitalization preceding the nursing home admission.

Data elements evaluated as potential DRT explanatory variables included: gender, age, race/ethnicity, time since last dialysis, whether the scheduled dialysis session preceding the index dialysis was missed, dialysis treatment frequency in the prior week, pre‐HD systolic blood pressure (sBP), peridialytic sBP change, and intradialytic hypotension (IDH) during the index dialysis session.

The race/ethnicity variable was built sequentially by first assigning ethnicity and then designating the non‐Hispanic population as White, Black, or other/unknown.

Nurses recorded missed dialysis sessions contemporaneously in the EMR for patients with an active prescription and a scheduled dialysis session who did not present for treatment, usually due to patient refusal, outside appointment, because the patient was deemed too medically unstable to receive treatment, or local administrative failure to produce the patient for care.

All vital signs were collected routinely during standard delivery of care. Peridialytic sBP changes were calculated as post‐HD sBP minus pre‐HD sBP, with a change of at least +5 mmHg constituting an increase in the dichotomous variable.

Intradialytic hypotension (IDH) is defined according to Flythe with modification,^19^, ^20^, ^21^ using a threshold absolute nadir sBP <90 mmHg during a dialysis session, or alternatively, nadir sBP <100 mmHg during a dialysis session for which pre‐HD sBP is ≥150 mmHg. For every dialysis session, we assessed whether IDH was observed.

Duration of dialysis vintage was obtained from the vintage field in EMR where there is considerable incompleteness. Length of stay in the SNF was calculated for those uncensored by study end date.

Those patients who did not meet the inclusion criteria were designated as the excluded cohort. Those excluded either never reported a recovery time, or their recovery time report lacked the required full week dialysis treatment opportunity in the antecedent week. We describe the excluded cohort to assess the internal generalizability of our findings to the entire SNF population. To build this comparison, descriptive data were obtained from the first “eligible dialysis” session of each patient during their first admission. For the DRT cohort, the first eligible dialysis session was defined as the first dialysis treatment after the patient had a full Monday to Sunday opportunity for receipt of treatment following the initiation of a dialytic episode in the SNF. For patients whose dialytic episodes began before November 4, 2011, this session could occur several weeks into the admission. For the excluded cohort, this constituted the first dialysis received in the SNF after November 4th, 2019.

This study adhered to the principles outlined in the Declaration of Helsinki. The Institutional Review Board (Western IRB) ruled this study protocol exempt under 45 CFR § 46.104(d) (2) of the Common Rule with a full waiver of HIPAA authorization for use and disclosure of protected health information for this research.

### Statistical method

Stata 17 was used for data management, descriptive statistics, mixed model logistic regression, and survival analysis. The mixed model logistic regression used a separate constant random effect at the patient level, as individual patients reported recovery time over multiple dialysis sessions. Explanatory variables were chosen in advance of model building and are included in the results even when not significant, as we deemed them biologically plausible at the outset of the study. The outcome variable, recovery within 2 h, was chosen in advance of model building.

Summary descriptions are provided as a modified Tukey 5‐point data summary (FPDS)^22^ with 5, 25, 50, 75, and 95 percentiles. This exclusion of extremes prevents inferential error due to gross database outliers.^22^ In tables, missing data are implied by differing counts of relevant constituent observations. Problem list diagnostic codes were aggregated into Charlson diagnostic categories^23^, ^24^, ^25^ using the Stata routine, Charlson (V. Stagg).^26^ Proportions were analyzed by chi‐square statistics, and continuous variables were compared with a t‐test. Instances of EMR‐recorded sBP ≥250 mmHg were considered erroneously recorded and were treated as missing values.

Ninety‐day survival analysis with outcome of death or hospitalization divided each dialytic episode into subintervals started by a dialysis session with an associated recovery attestation and ending with either another dialysis session with recovery time or dialytic episode terminating event.^27^, ^28^ We thereby produced two synthetic cohorts with consistent DRT for graphical display and significance testing using Logrank and Cox proportional hazards model.^29^

## RESULTS

From November 4, 2019 to June 11, 2021, there were 253,267 dialysis treatments provided, with 153,140 (60.5%) queried regarding recovery time and 136,280 (53.8%) providing answers (Table 1). The remaining 16,860 (6.7%) treatments involved patients with cognitive or physical impediments that prevented the collection of DRT information.

**TABLE 1:** Recovery time reports in all dialysis sessions

Recovery time category	Dialysis sessions (% total) (*n* = 253,267)	Cumulative percent of dialysis sessions with recovery reports (*n* = 136,280)
0–½ h	81,020 (32.0%)	59.5%
½–1 h	26,760 (10.6%)	79.1%
1–2 h	17,513 (6.9%)	91.9%
2–4 h	5835 (2.3%)	96.2%
4–8 h	1689 (0.7%)	97.5%
8–12 h	1305 (0.5%)	98.4%
By next morning	1701 (0.7%)	99.7%
Not even by next morning	457 (0.2%)	100%
N/A—cognitive deficit	16,105 (6.4%)	
N/A—physical impediment	755 (0.3%)	
Not asked/recorded	100,127 (39.5%)	

Abbreviation: N/A, not applicable.

The DRT cohort consisted of 2309 patients meeting the inclusion criteria with recovery reports in 108,876 dialysis sessions. The excluded cohort comprised 2489 patients, of whom 590 (24%) had recovery time information on 27,404 dialysis sessions.

Clinical and demographic descriptions of the first eligible dialysis for each patient in the DRT and excluded cohorts are provided in Table 2.

**TABLE 2:** Clinical description of first eligible dialysis in DRT and excluded cohorts

	DRT (*n* = 2309)							Excluded (*n* = 2489)						
	*n* (%)	Mean (SD)	Percentiles					*n* (%)	Mean (SD)	Percentiles				
			5th	25th	50th	75th	95th			5th	25th	50th	75th	95th
Age	2309	68 (12)	46	61	69	76	86	2489	69 (12)	48	63	71	78	87
Gender														
Female	1090 (47%)							1164 (47%)						
Male	1219 (53%)							1325 (53%)						
Race/ethnicity														
White	927 (40%)							981 (39%)						
Black	696 (30%)							916 (37%)						
Hispanic	204 (9%)							221 (9%)						
Other/unknown	482 (21%)							371 (15%)						
Vintage (years)	1477	2.5 (3.5)	0	0	1	5	9	1891	5.6 (14.6)	0	0	1	5	11
IDH during the reported dialysis	408 (18%)							390 (24%)						
sBP Increase (PostHD sBP—PreHD sBP) >5	902 (39%)							1046 (42%)						
Pre‐HD sBP	2309	131 (29)	98	111	129	150	178	2489	127 (28)	90	109	124	144	175
Post‐HD sBP	2309	130 (26)	98	111	127	146	174	2489	127 (27)	93	111	125	143	174
Net fluid removed (L)	2225	0.87 (0.82)	−0.3	0.2	0.8	1.4	2.2	2460	0.89 (2.4)	−0.3	0.2	0.7	1.2	2.2
UFR (ml/h/kg)	1880	4.54 (3.3)	0	2.25	4.22	6.47	9.80	2108	4.9 (12.3)	0	2.1	3.9	6.3	9.7
Length of stay (days) (uncensored)	2060	71 (102)	15	22	35	72	246	2329	35 (76)	3	8	14	28	140
Charlson index score	2269	3.6 (2)	2	2	3	5	7	2011	3.9 (2)	1	2	3	5	7
Acute MI	69 (3%)							74 (4%)						
CHF	676 (30%)							592 (29%)						
PVD	256 (11%)							192 (10%)						
Cerebrovascular	292 (13%)							242 (12%)						
Dementia	114 (5%)							66 (3%)						
COPD	384 (17%)							298 (15%)						
Rheumatologic disease	17 (0.7%)							44 (2%)						
Peptic ulcer	14 (0.6%)							28 (1%)						
Liver (mild)	84 (4%)							70 (4%)						
Liver mod/severe	17 (0.7%)							12 (0.6%)						
Diabetes	509 (22%)							419 (21%)						
Diabetes with complications	599 (26%)							383 (19%)						
Hemiplegia/paraplegia	19 (0.8%)							9 (0.4%)						
Renal disease	2112 (93%)							1828 (91%)						
Cancer	85 (3.7%)							103 (5%)						
Cancer with Metastasis	6 (0.3%)							8 (0.4%)						
AIDS	18 (0.8%)							12 (0.6%)						
Primary access	2300							2249						
HD catheter tunneled	1257 (55%)							1266 (56%)						
AV fistula	749 (33%)							721 (32%)						
AV Graft	255 (11%)							206 (9%)						
Miscellaneous	39 (2%)							56 (2%)						
Outcome of dialytic episode	2309							2489						
Censored	249 (11%)							160 (9%)						
Death	225 (10%)							282 (11%)						
Discharge														
Hospital	1009 (44%)							1109 (45%)						
Home	483 (21%)							503 (20%)						
Unspecified	286 (12%)							378 (15%)						
HD Withdrawn or patient recovered	57 (3%)							57 (2%)						

Abbreviations: AIDS, acquired immunodeficiency syndrome; AV, arteriovenous; CHF, congestive heart failure; COPD, Chronic obstructive pulmonary disease; DRT, post‐Dialysis Recovery Time; HD, hemodialysis; IDH, intradialytic hypotension; MI, myocardial infarction; PVD, peripheral vascular disease; sBP, systolic blood pressure; SD, standard deviation.

Compared to the excluded group, the DRT cohort was younger by 1.7 years (95% Confidence Interval [CI] [−2.4, −1.0]; *p* < 0.001), had shorter dialysis vintage by 3.2 years (95% CI [−3.9, −2.4]; *p* < 0.0001), longer SNF dwell time before the first reported recovery by 12.6 days (IQR 9.9–15.3; *p* < 0.001), and less IDH (18% vs. 24%, *p* < 0.001) (Table 2).

Weekly treatment frequency intent in the DRT cohort, as indicated by weekly dialysis prescriptions, varied over the course of the study. 74%–85% of patient‐weeks had 5x per week prescriptions and 85%–96% had 4x or 5x per week prescriptions—demonstrating a high prevalence of MFD intent. The actual weekly dialysis experience was a median of five treatments per week (IQR: 4–5) and a mean of 4.3 (SD 0.96). Median missed treatments was 0 (IQR: 0–1).

## DRT OUTCOMES

Of the DRT cohort in their first eligible dialysis session, 1838 patients (79.6%) reported recovery to baseline in ≤1 h and 2118 (91.7%) patients reported recovery to baseline in ≤2 h. This dialysis session occurred a median of 12 (IQR: 11–14) days after the first SNF dialysis that defined the beginning of the dialytic episode.

Ultrafiltration rate in the DRT cohort was median 4.6 ml/h/kg.

**FIGURE 1:**
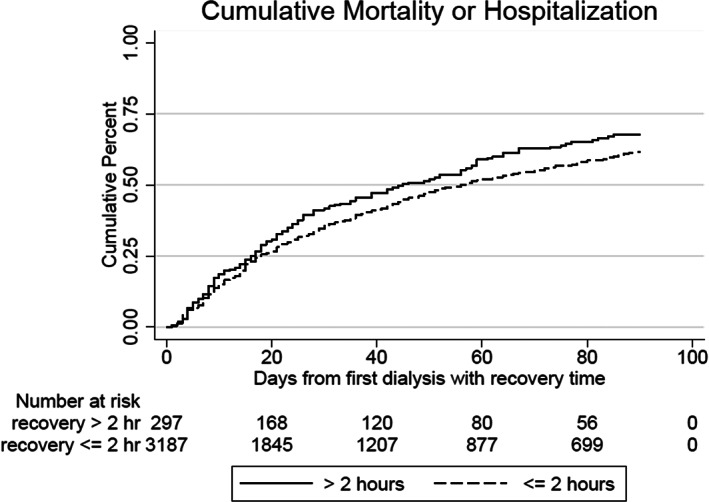
Cumulative incidence of mortality or hospitalization from the first dialysis with recovery time attestation through day 90, comparing synthetic patients with constant recovery time ≤2 h versus synthetic patients with constant recovery time >2 h. Rapid recovery (≤2 h) was associated with reduced mortality or hospitalization (*p* = 0.04). Analysis was performed on dialytic episodes with recovery time subintervals defined according to Anderson–Gill methodology

Cumulative mortality or hospitalization from the first qualifying dialysis through day 90 was significantly lower for recovery time ≤2 h (*p* = 0.04) (Figure 1).

In the excluded cohort, of 590 patients who did report recovery time, 477 (81%) reported recovery to baseline in ≤1 h and 490 (92%) reported recovery to baseline in ≤2 h in their first recovery time assessment. 570 (97%) of these recovery times were reported about a dialysis session within 3 days of the patient's first in‐SNF dialysis.

### Variables impacting DRT

Statistical modeling of rapid recovery time (≤2 h) in the DRT cohort revealed lower odds of rapid recovery time in older patients, in those who missed their prior prescribed dialysis session, and in those who experienced IDH (Table 3). Greater odds of rapid recovery were observed for patients who received five dialysis treatments in the week antecedent to the index dialysis and for those with pre‐HD sBP in the range of 160–179 mmHg. Hispanic race/ethnicity was associated with rapid recovery time, but there was no association with gender. The Intraclass Correlation Coefficient (patient random effect) was 0.76 (95% CI 0.73–0.78).

**TABLE 3:** Odds of rapid recovery time ≤2 h by mixed model logistic regression (*n* = 2309)

Variable	Odds ratio (95% confidence interval)	*p* value
Gender		
Male	1 (base)	
Female	0.89 (0.68, 1.2)	0.4
Age category		
0–50	2.2 (1.3, 3.7)	0.002
50–60	0.98 (0.70, 1.4)	0.9
60–70	1 (base)	
70–80	1.1 (0.81, 1.4)	0.6
≥80	0.97 (0.65, 1.5)	0.9
Race/ethnicity		
White	1	
Black	0.81 (0.59, 1.1)	0.2
Hispanic	2.4 (1.4, 4.1)	0.001
Other/unknown	1.3 (0.91, 2.0)	0.14
Number of dialyses in the antecedent week		
<5	1 (base)	
≥5	1.2 (1.1, 1.3)	0.000
Intradialytic hypotension	0.91 (0.84, 0.99)	0.025
Post‐HD—Pre‐HD sBP >5 mmHg	1.03 (0.96, 1.1)	0.43
Time since last treatment (days)	1.02 (0.99, 1.1)	0.24
Missed antecedent dialysis	0.73 (0.64, 0.83)	0.000
Pre‐HD sBP category		
0–119	0.95 (0.88, 1.03)	0.22
120–139	1 (Base)	
140–159	1.02 (0.93, 1.1)	0.72
160–179	1.2 (1.07, 1.4)	0.002
≥180	0.97 (0.80, 1.15)	0.71

Abbreviations: HD, hemodialysis; sBP, systolic blood pressure.

To assess potential baseline differences between race/ethnicity groups, we reviewed several characteristics of the first dialysis session according to race/ethnicity. Of first dialyses with pre‐HD sBP within 160–179 mmHg, 19% (63/332) were identified as Hispanic, 12% (136/1228) Black, 10.6% (153/1448) White, and 12.5% (72/576) other. Age and gender were similar across race/ethnicity.

To perform a sensitivity analysis of DRT only in the oldest patients, we reran our statistical model excluding all patients aged <50. Except for the loss of statistical significance for the IDH variable as a DRT predictor, all other findings in Table 3 were substantively unchanged (Table S1).

## DISCUSSION

Lindsay et al. demonstrated that time to recovery after HD is a reliable and validated health quality of life measure.^1^ Subsequent studies established correlations with other quality of life measures and physical symptoms scores.^2^, ^3^, ^4^, ^5^

Of the 2309 SNF dialysis patients we evaluated for statistical modeling with a mean age of 68, 80% reported resolution to baseline within 1 h, and 92% reported resolution to baseline within 2 h. We searched the literature for studies on post‐dialysis recovery time which report their findings as a proportion of patients recovering to baseline within 1 h and/or within 2 h. These metrics are easily understood and should be functionally useful for administrative determination of readiness for activities such as rehabilitation therapy—a common nursing home activity. We avoided those articles providing DRT solely as means and/or medians of minutes to recovery. This latter metric requires assigning numerical values to interval reports such as the “half a day” answer category, resulting in illusory precision, but more importantly this provides a less helpful summary when the objective is to determine the proportion of patients “ready” for SNF activities after a certain amount of time following treatment. DRT ≤2 h in patients receiving conventional 3x/week dialysis has generally been reported in a range of 21%–61.6% (Table 4). Two outlier studies by Lopes^2^ and Leme^32^ with young Brazilian patients (mean ages 49 and 50.5, respectively) reported <2 h recovery times of 93% and 83.2%, respectively.

**TABLE 4:** Conventional (3x/week) dialysis literature summary: Percent post‐dialysis recovery time (DRT) <1 and <2 h

Study	*n*	Study/data type	% DRT <1 h	% DRT <2 h	Age (mean (SD))
Guedes 2020^10^	98,616	Observational study of Incident patients (North America LDO).^a^ Annual KDQOL survey	44.3%	61.6%	62.6 (14.4)
Rayner 2014^6^ (DOPPS)	6040	Random sample (12 Western countries)	—	32.1%	64.8 (14.0)
Hussein 2017^30^	2689	Cross‐sectional study (46 US facilities, 3 states)^b^	—	55%	63 (median)
Lopes 2014^2^ (PROHEMO)	800	Cross‐sectional study (Brazilian patients)	84.9%	93.0%	49.0 (13.9)
Davenport 2018^3^	701	Cross‐sectional study (5 UK dialysis centers)^c^	24%	—	64.1 (16.6)
Harford 2017^31^	364	Prospective cohort (includes 1572 sessions, 3 US facilities)	—	52.1%	59 (median)
Alvarez 2020^7^	359	Cross‐Sectional study (NKF survey database)^c^	21%	39%	62.5 (13.8)
Leme 2020^32^ (HDFIT)	176	Cross‐sectional study (South/Southeast Brazil)^c^ ^,^ ^d^	73.8%	83.2%	52.5 (14.9)
Jaber 2010^15^ (FREEDOM)	128	Prospective Cohort measured at baseline on Conventional HD (US, Multicenter)	19.0%	—	52.0 (15.0)
Bossola 2013^33^	100	Cross sectional study (single site in Lazio, Italy)^e^	—	21%	61.7 (16.2)
Ozen 2021^9^	86	Cross‐sectional study (Single site in Turkey)^e^	19.8%	26.8%	56.4 (14.2)
Garg 2017^4^	86	Conventional HD Arm of Daily Trial RCT: Baseline & 12‐month Per Protocol assessment. (Multicenter in US and Canada)	20.5% (baseline), 37% (12 months)	—	50.4 (13.9)
Brys 2020^34^	45	Cross‐sectional study (single site, Rome, Italy)^f^	—	53.3%	63.0 (17.0)

*Note*: Minimum dialysis vintage criteria are not specified in study methods unless otherwise indicated.

Abbreviations: DOPPS, dialysis outcomes and practice patterns study; HD, hemodialysis; HDFIT, Impact of Hemodiafiltration on Physical Activity and Self‐reported Outcomes: A Randomized Controlled Trial; KDQOL, kidney disease quality of life; LDO, large dialysis organization; NKF, national kidney foundation; PROHEMO, prospective study of the prognosis of chronic hemodialysis patients.

^a^ Dialysis vintage <180 days.

^b^ Dialysis vintage >60 days.

^c^ Dialysis vintage >3 months.

^d^ Dialysis vintage <24 months.

^e^ Dialysis vintage >6 months.

^f^ Dialysis vintage >1 year.

Reports comparing 3x/week vs. 6x/week dialysis suggest MFD shortens recovery time. Garg et al.,^4^ reporting on the Frequent Hemodialysis Network's randomized controlled trial,^13^ found 20.5% <1 h DRT at baseline on conventional HD. In the per protocol analysis at 12 months, those randomized to 3x/week treatment reported 37% <1 h, while those randomized to in‐center 6x/week treatment reported 57% <1 h—a dramatic improvement. The observational Freedom Trial^15^ found a similar improvement following a switch to 6x/week MFD.

To test the internal validity of our reported recovery times, we modeled the responses in a mixed linear logit model using variables we considered biologically plausible to impact recovery time. The outcome measure was rapid recovery time (defined as DRT ≤2 h), and the model provides relative odds for the variable's impact, with odds ratios greater than 1 indicating the variable increases the proportion of patients with rapid recovery. Intradialytic hypotension was expected to tax the patient's system and reduce the proportion experiencing rapid recovery. Consistent with these expectations and some prior studies,^9^, ^10^, ^30^ IDH was associated with reduced rapid return to baseline with 0.91 odds ratio (CI: 0.84–0.99; *p* = 0.025) (Table 3). We expected patients missing their previous dialysis session to be less likely to have short DRT. Concordantly, the missed antecedent dialysis variable resulted in an odds ratio of 0.73 (CI: 0.64–0.83; *p* < 0.001).

Similarly, we expected that a patient receiving five dialyses in the antecedent week would have better recovery time for two reasons. First, receipt of consistent dialysis on a frequent schedule prior to the index dialysis session should minimize biochemical and fluid derangements. Second, patients completing five dialyses in the antecedent week are more likely to be relatively healthy since we often see therapy declined by patients who complain of frailties. The observed odds ratio of 1.2 (95% CI, 1.1–1.3, *p* < 0.001) confirms our expectation.

Because all these variables could not have been in the consciousness of the nurse recording the patient's recall of the previous dialysis session's recovery time, the consistency between biological expectation of DRT variability and the observed results is a good indicator of construct validity. The finding of association between pre‐HD sBP in the 160–179 mmHg range and reduced recovery time is consistent with the impressions of nursing staff that higher initial BP is a “safer” blood pressure to dialyze with fewer complications (personal communication). The finding of better recovery time in Hispanics is unexplained by gender or age differences, but we did observe a higher proportion of Hispanics (19% vs. 11%) with pre‐HD sBP in the range 160–179 mmHg, which conferred higher odds of rapid recovery time. Of note, gender was not a statistically significant influence on recovery, a finding that agreed with Bossola^33^ and Awuah^35^ but contrasts with reports by other researchers.^6^, ^9^, ^10^, ^30^, ^36^

The Intraclass Correlation Coefficient of 0.76 (95% CI, 0.73–0.78) indicates that 76% of the variability in proportion of patients with recovery ≤2 h is explained by a tendency for individual patients to have a consistent experience and/or report of recovery. Patients tended to report a consistent recovery time across multiple surveys—a finding that intuitively makes sense, as we can imagine a characteristic frailty, experience, perception, and/or reporting inclination, which might be patient idiosyncratic. This is also consistent with previous data in conventional HD on a much smaller scale, where DRT was highly correlated across only three treatments in 267 and 364 patients, respectively.^31^, ^35^ The same studies, however, failed to find a relationship between time to recovery and age or IDH, a failure we attribute to their small sample sizes, few sampled events (only three surveys across successive dialyses or within 7 days per patient), and in one case analysis with a linear model, rather than a logistic model with a meaningful cut‐point.

Reduced cumulative mortality or hospitalization was associated with shorter recovery time (≤2 h), consistent with a similar relationship demonstrated previously^6^, ^10^ and providing further evidence that DRT reported in this study retained biologic plausibility. Our study is the first to demonstrate this relationship in the SNF, on‐site, MFD‐treated ESRD population. If the association between decreased DRT and reduced mortality/hospitalization is causal, reducing DRT has the potential to impact the patient beyond merely quality of life considerations.

The biological explanation for notably fast DRT in this cohort and the relatively high proportion of patients with rapid recovery compared to prior MFD studies could be the ultrafiltration rate (UFR). Correlation between DRT and UFR was identified previously.^6^, ^30^, ^33^ In the Frequent Hemodialysis Network study, UFR in the MFD group was 10.6 ml/h/kg (calculated from their Tables 1 and 2).^13^ In our SNF patients receiving MFD, median UFR was 4.9 ml/h/kg,^18^ and in the population modeled for DRT outcome in this study, UFR was 4.6 ml/kg/h. Patients living at home have unlimited access to salt and fluids, whereas in the SNF, access to discretionary salt and fluid is reduced, which significantly reduces intradialytic weight gain and consequently the dialytic fluid removal requirement. Combining lower fluid removal requirement with more frequent dialysis results in UFR <5 ml/h/kg, providing a “gentler” dialysis with a shorter post‐dialysis recovery time.

### Limitations

We purposely restricted our primary analysis to patients who were in the SNF long enough to represent the effect of SNF MFD dialysis routine rather than the consequences of a recent hospitalization. This excluded those with a recovery survey only very early in their nursing home stay. We expected proximity to hospitalization would result in early dialysis experiences with longer recovery times. However, we were surprised to find recovery ≤2 h in 92% of 590 patients in the excluded cohort, 97% of whose reports were within 3 days of SNF admission.

Either the DRT‐lowering effect of MFD in the SNF is more immediate than expected, occurring within the first few treatments of the nursing home stay, or self‐reported recovery time to baseline is suspect in all SNF‐resident patients. To our knowledge, the speed of onset of this DRT improvement has never been evaluated.

Certainly, any analysis reliant upon individual perception and recall is subject to validity questions. Furthermore, it remains unknown to what extent patient perception and therefore report of “return to baseline” is comparable between our population of sedentary, frail nursing home patients and that of the general dialysis population who must take public transportation, commute to a job, and take care of a home.

Even if imperfectly comparable, the reported findings would be valid from the SNF patient's perspective and could be relevant for important activities in the SNF requiring his/her active participation and general feeling of well‐being. In the future, we plan to study the impact of our dialysis methodology on rehabilitation participation—an important consideration for a population for whom ~25% of nursing home stays end in a return to home.^18^ This will serve as a real‐world test of the reported recovery time metric's applicability beyond perception. Another limitation to the study is the possible bias inherent in information collected by the nurse or dialysis technician. Since many patients received their dialysis in a dialysis den separate from their SNF bedroom, the nurses might interpret a release of the patient from the den without achieving baseline status as a poor reflection on their practice, and this could conceivably influence the nurses' recording of the patient's DRT report with a self‐interest toward protection from criticism of their work. The observed sensitivity of DRT to plausible biologically relevant causal factors not apparent to the recording nurses/technicians somewhat reduces this concern.

## CONCLUSION

Skilled nursing facility dialysis patients receiving 5x/week HD report rapid recovery time ≤2 h in 92% of dialysis sessions despite advanced age, frailty, and high comorbidity burden. Future studies are being planned to explore the functional import of short DRT for nursing home subacute and long‐term rehabilitation programs that could additionally have an impact on health extending beyond discharge from the SNF.

## CONFLICT OF INTEREST

AMK, AMH, and JGL are current employees of Dialyze Direct and hold stock and/or stock options in Dialyze Direct. SMK is a current employee of Dialyze Direct. NWL is a consultant for Dialyze Direct and chair of its Medical Advisory Board. EYB is a consultant epidemiologist for Dialyze Direct, and WTM is a consultant research analyst and medical writer for Dialyze Direct.

## Supporting information


Table S1
