# Supplementary material for: Post‐dialysis recovery time in ESRD patients receiving more frequent hemodialysis in skilled nursing facilities
Source: Hemodial Int. 2022 Apr 6;26(3):424–34. doi: 10.1111/hdi.13012 (PMC9543222; doi:10.1111/hdi.13012)
Supplement: Supplementary file 1 — Table S1 [file HDI-26-424-s001.docx]

**Post-Dialysis Recovery Time in ESRD Patients Receiving More Frequent Hemodialysis in Skilled Nursing Facilities**

Eran Y Bellin, Alice M Hellebrand, Steven M Kaplan, Jordan G Ledvina, William T Markis, Nathan W Levin, Allen M Kaufman

**Supplementary Material Table of Contents**

**Supplemental Table S1**: Sensitivity Analysis: DRT Statistical modeling including only patients ≥50 years old

**Supplemental Table S1.** Odds of Rapid Recovery Time ≤ 2 hours by Mixed Model Logistic Regression, including only patients aged ≥ 50 (n=2146)

| **Variable** | **Odds Ratio (95% Confidence Interval)** | ***p* value** |
| --- | --- | --- |
| **Gender**  Male  Female | 1 (Base)  0.85 (0.64, 1.12) | 0.25 |
| **Age Category**  50-60  60-70  70-80  ≥ 80 | 1 (Base)  0.87 (0.61, 1.2)  0.91 (0.62, 1.3)  0.83 (0.52, 1.3) | 0.44  0.64  0.42 |
| **Race/Ethnicity**  White  Black  Hispanic  Other/Unknown | 1  0.79 (0.57, 1.1)  2.3 (1.3, 4.1)  1.3 (0.87, 2.0) | 0.17  0.003  0.20 |
| **Number of dialyses in the antecedent week**  < 5  ≥ 5 | 1 (base)  1.21 (1.11, 1.31) | 0.000 |
| **Intradialytic Hypotension** | 0.94 (0.86, 1.02) | 0.13 |
| **Post-HD – Pre-HD sBP > 5 mmHg** | 1.04 (0.97, 1.11) | 0.29 |
| **Time Since Last Treatment (days)** | 1.02 (0.98, 1.05) | 0.30 |
| **Missed Antecedent Dialysis** | 0.75 (0.66, 0.86) | 0.000 |
| **Pre-HD sBP Category**  0-119  120-139  140-159  160-179  ≥ 180 | 0.96 (0.88, 1.04)  1 (Base)  1.03 (0.94, 1.14)  1.22 (1.08, 1.38)  1.00 (0.84, 1.20) | 0.30  0.49  0.002  0.97 |

HD, hemodialysis; sBP, systolic blood pressure.
